# Assessment of anemia and associated risk factors among children under-five years old in the West Guji Zone, southern Ethiopia: Hospital-based cross-sectional study

**DOI:** 10.1371/journal.pone.0270853

**Published:** 2022-07-05

**Authors:** Alqeer Aliyo, Abdurezak Jibril

**Affiliations:** 1 Department of Medical Laboratory Science, Bule Hora University, Bule Hora, Ethiopia; 2 Department of Nursing, Bule Hora University, Bule Hora, Ethiopia; UCSI University, MALAYSIA

## Abstract

**Background:**

Anemia adversely affects children’s mental, physical and social development, particularly in Africa. In the early stages of life, it leads to severe negative consequences on the cognitive, growth and development of children.

**Objective:**

This study aimed to assess anemia and associated risk factors among children under-five years old in the West Guji Zone, southern Ethiopia, from October to November 2020.

**Method:**

A hospital-based quantitative cross-sectional study was conducted at Bule Hora General Hospital, Southern Ethiopia. A convenience sampling technique was used to include 375 under-five children enrolled in the study. The pretested structure questionnaire was used to collect socioeconomic and demographic characteristics of study individuals after taking appropriate written informed consent. Then, a venous blood sample was collected from each child and analyzed for hemoglobin determination using a Midray BC 3000 Plus machine. Binary logistic regression models were used to identify associated factors of anemia. A p-value ≤ 0.05 was considered statistically significant.

**Result:**

The overall prevalence of anemia among under-five children was 13.2% (50) [95% CI = 5.2–21.2%]. Among anemic children under-five years of age, 12% (6) had mild anemia, 32% (16) had moderate anemia and 56% (28) had severe anemia. In this study, anemia was significantly associated with a history of intestinal protozoan infection [AOR = 2.55, 95% CI = 1.28–10.42], malaria infection [AOR = 5.01, 95% CI = 0.18–11.44] and soil-transmitted helminths infection [AOR = 6.39, 95% CI = 1.75–29.08].

**Conclusion:**

The prevalence of anemia among under-five children was found to be low in the study area; however, the majority of anemic children were in a severe stage. It could be managed by preventing malaria infection, intestinal protozoa and soil-transmitted helminthic infection.

## Introduction

Anemia is a condition that causes a decline in erythrocyte concentration in circulation or hemoglobin in the blood and a concomitant impairment of oxygen transportation [1]. The World Health Organization (WHO) defined anemia as hemoglobin (Hgb)<12 g/dL in adult nonpregnant women, Hgb <11 g/dL in pregnant females, Hgb <13 g/dL in adult men, Hgb <11 g/dl in children aged 6–59 months, Hgb <11.5 g/dl in children aged 5–11 years, Hgb<12 g/dl for children aged 12–14 years and Hgb <13 g/dL in newborns [2, 3].

Globally, 1.3 billion individuals suffer from anemia, making it one of the most important public health issues on the international agenda [4]. Globally, on average, approximately 9.6 million children are severely anemic [5]. It affects people in both developing and developed countries [6]. By 2017, 293.1 million (47.4%) children under-five years of age were anemic worldwide, and 67.6% of these children lived in Africa [1]. In Ethiopia, 57% of children aged 6–59 months are anemic according to the Ethiopian Demographic and Health Survey (EDHS) report [7].

Iron deficiency is the major cause of anemia in developing countries and results in insufficient red blood cell production. In some individuals, infections such as peptic ulcers may cause blood loss and anemia. In developing countries, iron deficiency impacts all vulnerable groups. Additionally, geographically specific infections such as malaria and helminthic contribute to excessive red blood cell destruction and cause excessive red blood cell loss. Other infectious diseases may also be at play [8].

Anemia impairs mental, physical, and social development and causes negative behavioral and cognitive effects, resulting in poor school performance and work capacity in later years [9]. In early childhood, poor feeding habits, especially during the weaning period, exacerbate the problem. Anemia frequently develops as breast milk is replaced by foods that are poor in iron and other nutrients, including vitamin B12 and folic acid. Low oxygenation of brain tissues, a consequence of anemia, may lead to impaired cognitive function, growth and psychomotor development, especially in children. Infants, children under 5 years old and pregnant women have greater susceptibility to anemia because of their increased iron requirements due to rapid body growth and expansion of red blood cells [9]. Moreover, anemia leads to immune system compromise, resulting in a decreased ability to fight infections and increased mortality in African children, where resources to determine the basic etiology remain poor [10, 11].

There are national and regional data on the prevalence of anemia and its risk factors among children under-five years of age in different parts of Ethiopia such as Gonder town (66.8%) [12], Wagmra zone, Ethiopia (66.6%) [13] and Shanan Gibe hospital 48.9% [14]. To our knowledge, no previous study has been undertaken in the study area of Bule Hora. Therefore, this study aimed to assess the assess of anemia and its associated factors among children from 6–59 months of age in Bule Hora Hospital, southern Ethiopia.

## Methodology

### Study area, period and design

The hospital-based quantitative cross-sectional study was conducted from October 26 to November 20, 2020, at Bule Hora General Hospital, Guji Zone: Oromia Region, southern Ethiopia. The hospital found in Bule Hora town 467 km from Addis Ababa capital city of Ethiopia. According to the Bule hora town municipality administrative, the current total population of Bule Hora town is 67,297. Geographically, the town is located between latitude 5°35’N and longitude 38°15’E and an altitude of 1716 meters above sea level. Regarding Bule Hora General Hospital, the hospital provides different services, including pediatrics, emergency, delivery, outpatient, patient, laboratory, pharmacy, medical and surgical services. Currently, the hospital has given the service for 5 million people in the area.

### Study population and selection

All children aged between 6 months and 59 months who attended Bule Hora General Hospital during the data collection period and voluntarily participated were included in the study. Children who were outside the range, had active bleeding and underwent surgery before one month were excluded from the study.

### Sample size and sampling technique

The sample size was determined using a single population proportion formula considering the prevalence of anemia from a previous study 66.8% [15].

$$\text{n}=\frac{Z^{2}p\left(1-p\right)}{d^{2}}=\;\frac{{\left(1.96\right)}^{2}*0.668\left(1-0.668\right)}{{\left(0.05\right)}^{2}}=341$$

where d = Margin of error between the sample and the population (d = 5%), n = Sample size, Z α/2 = 95% confident interval (1.96), P = 66.8% Prevalence. Then, a 15% nonresponse rate was added considering the response rate of a previous study; thus, the final sample size was **392**. A convenience sampling technique was used for all under-five children who fulfilled the inclusion criteria during the study period.

### Data collection instruments and procedure

The data were collected using a structured questionnaire adapted from previous literature [16]. The questionnaire was prepared in English in written form, orally translated into the Oromia and Amharic languages, and then returned to English to ensure its consistency. Five percent of the total sample respondents were interviewed during the pretest in another health institution. After this, the questionnaire was edited accordingly, and then the final version of the questionnaire was adapted to interview children’s parents/caregivers. The data collectors explained the objective of the study to the children’s parents/caregivers. Highlighting on the benefit of being tested for Hgb and what would be done if the child is anemic. The data collectors were given details to the patient/caregiver that no name of participant written on the questionnaire and confidentiality were protected, and verbal consent was obtained. Data were collected through pretested and structured questionnaires by face-to-face interviews with the children’s patients/caregivers. The questionnaire was used to collect sociodemographic data and associated factors [S1 File].

### Blood sample collection

By strictly following the standard operating procedure (SOPs), a 3 ml venous blood sample was collected. Experienced laboratory technicians collected the samples in tubes containing ethylenediaminetetraacetic acid (EDTA). The complete blood count (CBC) reports from the hematology analyzer (Midray BC 3000Plus manufactured by Jeevika, Chinese Company) in the hospitals, including hemoglobin (Hgb) analysis, were performed as per the manufacturer’s instructions.

### Operational definitions

Anemia—Hemoglobin (Hgb) <11 g/dL in children whose age is 6–59 months [17].

Mild anemia: The Hgb value is 10–10.9 g/dL [17].

Moderate anemia: Hgb value 7–9.9 g/dL for children 6–59 months [17].

Severe anemia: Hgb value <7 g/dL for children aged 6–59 months [17].

Low income: family monthly income less than 750 birr (ETB) [18].

Moderate income: family monthly income from 750–1500 ETB [18]

High income: family monthly income greater than 1500 ETB [18]

### Data quality control

To assure data quality, 5% of the estimated sample was pretested at the Bule Hora health center before the data collection to determine whether the questionnaires were simple and understandable. After every data collection, the completeness and consistency of the questionnaire were checked. Data collector training and daily supervision were performed before and during the data collection period.

During blood sample analysis, the standard operating procedures (SOPs) and manufacturers’ instructions were strictly followed for all laboratory activities. The sample was checked for hemolysis, clotting and enough volume before running the test. When the machine passed the control, the samples were analyzed. Finally, the laboratory test result was recorded, and the specimens were managed properly.

### Data processing and analysis

Data were entered, sorted and categorized. Data cleaning was performed to check for completeness, accuracy, and missed values, and any errors identified were corrected. Then, the data were analyzed using SPSS version 22. Descriptive statistics (mean, frequency) were carried out to describe the sociodemographic status of the participants presented by table, pie chart and graph. A binary logistic regression model was fitted to identify factors associated with anemia. Variables with a p-value ≤ 0.25 in the bivariate analysis were considered candidates for the multivariate analysis. Multivariate logistic regression was performed to control for possible confounding and identify the true effect of the selected predictor variables. The model fitness was checked with the Hosmer–Lemeshow test. The extant association between the different variables related to anemia was measured using AOR at 95% CI. A p-value ≤ 0.05 was considered statistically significant.

### Ethics approval and consent to participate

The study was conducted after ethical approval from the Bule Hora University Research and Ethical Review Committee. The official letter was written to Bule Hora General Hospital. Informed, voluntary, written signed consent was obtained from the study participant, parent/caregiver and institution. The children’s parents/caregivers were informed about the purpose of the study, and written informed consent was obtained before the questionnaire was administered. Then, blood samples were collected from the study participants. Participation in the study was voluntary. The participants were informed of their right to quite/refuse their participation at any stage of the study if they did not want to participate. To ensure confidentiality of participant information, codes were used, and any identifier of participants was not written on the questionnaire on the test tube. Any abnormal test results of participants were communicated to the concerned body.

## Results

### Sociodemographic and economic status

Of the 392 children under the age of five, 375 participated in this study, with a 95.7% response rate. The participants’ ages ranged from 6 to 59 months, with a mean (SD) age of 3.3 (± 2.20) months. Among 375 children under the age of five, 54.4% (204) were female, and the majority 39.7% (149) of children’s mothers/caregivers were farmers. More than half, 54.4% (204), were from rural areas. Approximately 29.4% (110) of child caregivers were unable to read and write. Out of 375 children under the age of five, approximately 55.9% (210) were protestants. Majority of children 67.7% (254) were living in a household less than 3 family members, and the majority of households 36.8% (138) had incomes less than 750 Ethiopian birr (Table 1).

**Table 1:** Sociodemographic and other selected characteristics of under-five children attending Bule Hora General Hospital, November 2020.

Variables	Category	Frequency	Percent
Age groups	6–23 months	138	36.80
	>23 months	237	63.20
Sex of child	Male	171	45.6
	Female	204	54.4
Religious	Orthodox	81	21.7
	Muslim	81	21.7
	Protestant	210	55.9
	Others	3	0.7
Sex of care giver	Male	204	54.4
	Female	171	45.6
Marital status of care giver/mothers	Married	281	75.0
	Divorced	33	8.8
	Widowed	11	2.9
	Single	50	13.2
Educational level of care giver/mothers	Unable to read and write	110	29.4
	Able to read and write	72	19.1
	Grade 1–8	88	23.5
	Grade 9–12	28	7.4
	College and above	77	20.6
Occupational of care giver	Herder	0	0.0
	House wife	77	20.6
	Merchant	39	10.3
	Farmer	149	39.7
	Private employee	44	11.8
	Government employee	66	17.6
Sex of household head	Male	292	77.9
	Female	83	22.1
Family size	≤3	254	67.7
	>3	121	32.3
Residence	Urban	171	45.6
	Rural	204	54.4
Income of care giver	< 750 ETB	138	36.8
	750–1500 ETB	127	33.9
	> 1500 ETB	110	29.3

#### Feeding-related factors

Regarding feeding practices, among 375 under-five children, 69.1% (259) reported dietary diversity practices, 79.4% (298) reported the introduction of complementary food after 6 months, 61.8% (232) reported that mothers/caregivers did not have nutritional knowledge, 23.5% (88) reported food insecurity within four weeks, 79.5% (298) reported eating three times and above per day, and the majority of 80.8% (303) reported breastfeeding practices at 6–12 months (Table 2).

**Table 2:** Feeding-related characteristics of children.

Variables	Category	Frequency	Percent
Dietary diversity Practice	Yes	259	69.1
	No	116	30.9
Product use in dietary diversity	Animal product	11	2.9
	Plant product	0	0.0
	Both	364	97.1
Introduction of complementary foods	≤6 months	77	20.6
	> 6 months	298	79.4
Duration of breast feeding practice	< 6month	28	7.5
	6-12month	303	80.8
	1<	44	11.7
Have nutritional knowledge	Yes	143	38.2
	No	232	61.8
Food insecurity in past four weeks	Yes	88	23.5
	No	287	76.5
Animal products use	Yes	303	80.9
	No	72	19.1
Meal frequency	≤2	77	20.5
	≥3	298	79.5

### Health care and disease characteristics

Out of 375 under-five children enrolled in the study, 17.6% (66) had a history of intestinal protozoa followed by soil-transmitted helminthic infection 16.2% (61) and malaria infection 14.7% (55) (Table 3).

**Table 3:** Clinical characteristics of children.

Variables	Category	Frequency	Percent
Recent acute blood loss	Yes	28	7.4
	No	347	92.6
Recent blood transfusion reaction	Yes	6	1.5
	No	369	98.5
Recent surgical procedure	Yes	0	0.0
	No	375	100.0
Recent accident injury	Yes	22	5.9
	No	353	94.1
Recent intestinal protozoa infection	Yes	66	17.6
	No	309	82.4
Recent helminthic infection	Yes	61	16.2
	No	314	83.8
Recent malaria infection	Yes	55	14.7
	No	320	85.3
Recent epistaxis	Yes	22	5.9
	No	353	94.1
Chronic diseases	Yes	11	2.9
	No	364	97.1

### Prevalence of anemia

**Fig 1:**
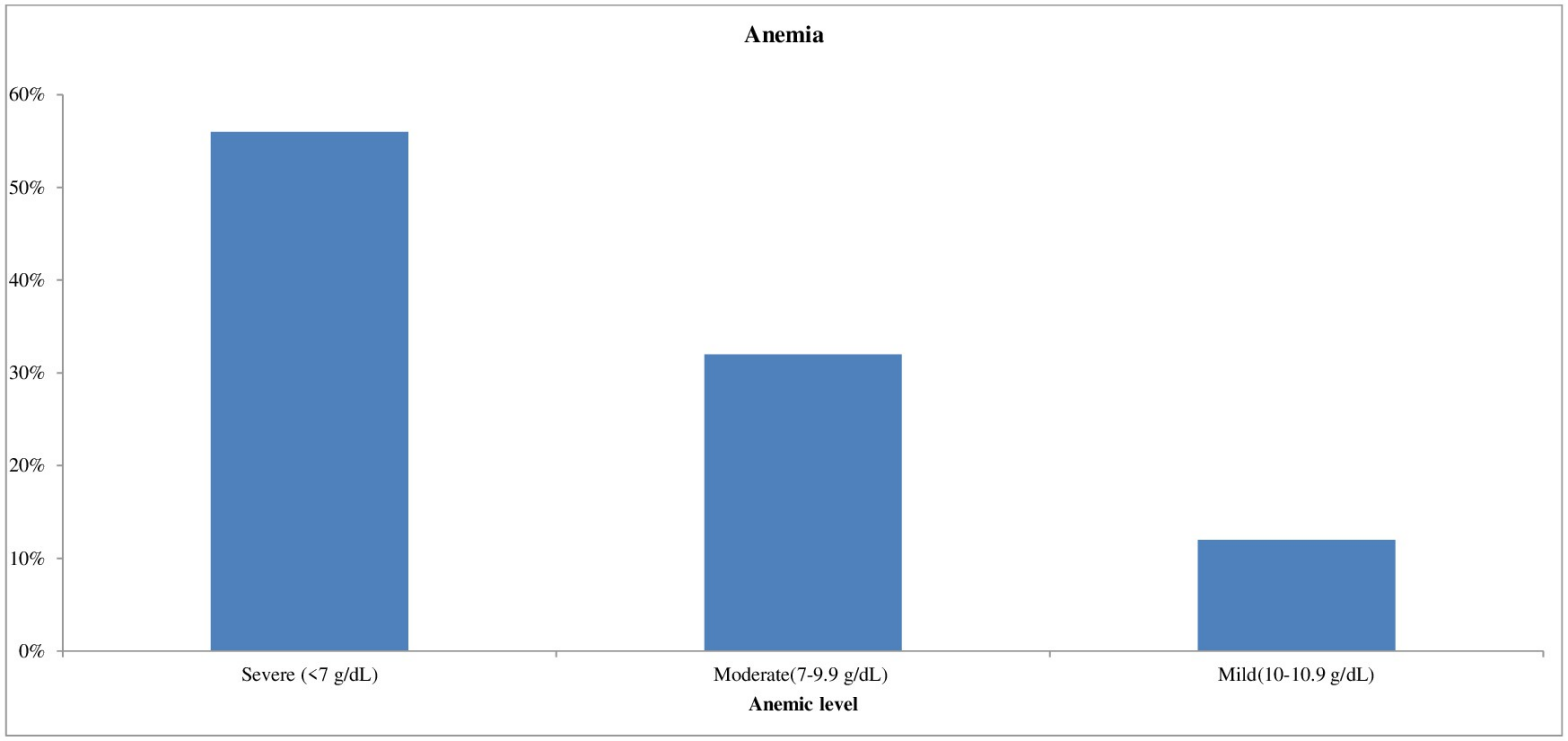
Bar chart that shows anemic level among under-five years old children, 2020. Anemic level among under-five year’s old children. Anemic children scores hemoglobin values of 10–10.9 g/dl were mild anemia, 7–9.9 g/dl were moderate anemia, and less than 7 g/dL were determined severe anemia.

Based on the hemoglobin cutoff value, less than 11 g/dL was categorized as anemic, and Hgb values of 10–10.9 g/dl, 7–9.9 g/dl, and less than 7 g/dL were determined to be mild, moderate and severe, respectively. The overall prevalence of anemia was 13.2% (50). Among anemic children under-five years of age, 12% (6) were mild, 32% (16) were moderate and 56% (28) were severe (Fig 1).

### Factor associated with prevalence of anemia

In bivariate analysis, variables such as child age group, child sex, family size, income of caregiver, dietary diversity practice, breastfeeding practice, nutritional knowledge, meal frequency, recent intestinal protozoan infection, recent helminthic infection, and recent malaria infection had a p-value of <0.25 and were considered candidates for multivariate analysis. In multivariate analysis, the chance of having anemia was approximately 3 times higher among children under the age of five who had a history of intestinal protozoan infection [AOR = 2.55, 95% CI = 1.28–10.42] than among their counterparts. Additionally, children who had a history of soil-transmitted helminths infections were 6 times more likely to have anemia [AOR = 6.39, 95% CI = 1.75–29.08] than their counterparts. Similarly, the children who had a history of malaria infection were nearly 5 times more likely [AOR = 5.01, 95% CI = 0.18–11.44] to have anemic than those who did not have recent malaria infection (Table 4).

**Table 4:** Bivariate and multivariate analyses of factors associated with the prevalence of anemia among under-five year, 2020.

Variables	Category	Anemic	Non anemic	COR(95%CI)	AOR(95%CI)
Age group	6–23 months	23(16.70%)	115(83.30%)	1.56(0.24–4.22)	0.94(0.08–2.05)
	>23 months	27(11.40%)	210(88.60%)	1	1
Child sex	Female	28(13.70%)	176(86.30%)	1.08(0.26–4.32)	1.35(0.43–7.63)
	Male	22(12.91%)	149(87.09%)	1	1
Family size	≤3	39(15.4%)	215(84.60%)	1.8(0.64–5.75)	2.12(0.88–6.11)
	>3	11(9.10%)	110(90.10%)	1	1
Income of care giver	< 750 ETB	28(20.30%)	110(79.70%)	0.71(0.15–3.40)	0.45(0.21–1.76)
	750–1500 ETB	6(4.70%)	121(95.30%)	3.88(0.37–4.71)	2.63(0.33–4.87)
	> 1500 ETB	16(14.50%)	94(85.50%)	1	1
Dietary diversity practice	Yes	39(14.90%)	220(85.10%)	1.66(0.32–8.78)*	1.98(0.41–7.55)
	No	11(9.50%)	105(90.50%)	1	1
Duration of breast feeding practice	< 6month	6(21.40%)	22(78.60%)	1	1
	6-12month	38(12.50%)	265(87.50%)	0.42(0.03–5.32)*	0.39(0.11–2.09)
	1<	6(13.60%)	38(86.40%)	3.88(0.37–21.71)**	3.63(1.05–11.00)
Have nutritional knowledge	Yes	17(11.50%)	126(88.50%)	1	1
	No	33(14.30%)	199(85.70%)	1.23(0.29–5.62)	1.07(0.02–5.36)
Meal frequency	≤2	17(22.08%)	60(77.92%)	2.28(0.45–7.11)	2.42(0.84–9.50)
	≥3	33(11.07%)	265(88.93%)	1	1
History of intestinal protozoa infection	Yes	11(16.70%)	55(83.30%)	1.38(1.13–7.98)**	2.55(1.28–10.42**
	No	39(12.60%)	270(87.40%)	1	1
History of soil helminthic infection	Yes	22(36.10%)	39(63.90%)	5.76(0.82–21.18)**	6.39(1.75–29.08)*
	No	28(8.80%)	286(91.20%)	1	1
History of malaria infection	Yes	22(40%)	33(60%)	6.95(1.03–14.75)***	5.01(0.18–11.44)*
	No	28(8.8%)	292(91.20%)	1	1

Statistical significance at P<0.001 = ***, P<0.01 = ** and at P<0.05 = *, COR = crude OR and AOR = adjusted OR with CI = confidence interval.

## Discussion

In this study, the overall prevalence of anemia among under-five children attending Bule Hora General Hospital was 13.2% (50) (95% CI = 5.2–21.2%). According to the WHO definition, anemia can be defined as a mild, moderate and severe public health problem when the prevalence is 5–19.9%, 20–39.9% and greater than 40%, respectively. Therefore, the prevalence of anemia in this study is considered a mild public health concern [19].

This is lower than the previous study conducted in Western China (51.2%) [20], Eastern Sudan (86%) [21], Cape Verde West Africa (51.8%), [22], Nigeria (70.5%) [23], Tanzania (77.2%) [24], Gonder town Ethiopia (66.8%) [12], Gonder, Ethiopia (58.6%), [25], Wagmra zone, Ethiopia (66.6%) [13], South Wollo, Northeast Ethiopia (41.1%) [18] and Shanan Gibe hospital 48.9% [14]. The difference in prevalence might be due to variations in the number of participants, sample analysis equipment, hemoglobin cutoff points, and cultural, geographical and behavioral characteristics of the community. In contrast, this finding was higher than reports from Brazil (10.2%) [26] and Ebonyi, Nigeria (9.7%) [27]. The possible reason might be due to the age difference of the study participants, and the children who were recently treated or who took iron supplements were not excluded from the study.

Multivariate analysis showed that having a previous intestinal protozoan infection was significantly associated with the prevalence of anemia among under-five children. This finding is in line with a study conducted in Pawe town, Benishangul Gumuz, region [28], Gonder, Ethiopia [25] and Shanan Gibe hospital [14]. Similarly, children who had previous malaria infection were significantly associated with anemia among under-five children. This finding agrees with a study conducted in Ghana [29]. In addition, anemia was 6 times more likely among children with previous soil-transmitted helminthic infections than among their counterparts. This finding is consistent with a study conducted in Gonder, Ethiopia [12]. Possible justification could be due to intestinal wall bleeding, erythrocyte lysis, reduction of iron absorption and damaged organs involved in hematopoiesis.

Out of 375 children under-five years of age involved in the study, nearly half (48.5%, 182) were infected with at least one of the following species of parasites: *Plasmodium spp*., *Entamoeba histolytical*, *Giardia lambia*, *Ascaris lumbricoides*, *Strongyloides stercoralis*, *Trichuris trichuria*, *Enterobius vermicularis* and *Hookworm*. Of those children infected with recent malaria, 22 (40%), soil-transmitted helminths, 22 (36.1%) and intestinal protozoa 11 (16.7%) were found to be anemic. The parasite species may deplete red blood cells through loss of blood at the time of diarrhea and gastrointestinal bleeding.

### Strength and limitations of the study

The strength of this study, it could elaborate the important part of health accessibility issues, especially in child health, and identified associated factors of anemia. Regarding the limitations of the study, as a convenience sampling technique was applied, the result cannot be extrapolated to a larger community. Respond bias may have been introduced during the interviews. Additionally, this study does not differentiate the types of anemia such as hemolytic, sickle cell and thalassemia.

## Conclusion

The present study demonstrated a 13.2% overall prevalence of anemia among children under the age of 5 who attended Bule Hora General Hospital. This finding was low compared with previous studies conducted in different parts of Ethiopia. The factors significantly associated with anemia were recent intestinal protozoan infections, soil-transmitted helminths, and malaria infection. Despite the limitations, where it highlights the necessity for early comprehensive care in diagnosis and treatment with attention of prevention program (deworming) for reduction of anemia among under-five children.

## Supporting information

S1 File Questionnaire. (PDF)

## Abbreviations

**AOR**: adjusted odds ratio
**CBC**: complete blood count
CI: confidence interval
**COR**: crude odds ratio
**EDHS**: Ethiopian Demographic Health Survey
**EDTA**: ethylene diamine tetraacetic acid
**ETB**: Ethiopian Birr
g/dL: gram per deciliter
HGB: hemoglobin
**SOPS**: standard operation procedures
**SPSS**: Statistical Package for Social Science
**WHO**: World Health Organization
