# Supplementary material for: Assessment of anemia and associated risk factors among children under-five years old in the West Guji Zone, southern Ethiopia: Hospital-based cross-sectional study
Source: PLoS One. 2022 Jul 5;17(7):e0270853. doi: 10.1371/journal.pone.0270853 (PMC9255756; doi:10.1371/journal.pone.0270853)
Supplement: S1 File — (PDF) [file pone.0270853.s001.pdf]

Annex II: Questionnaire

Date of interview	Date	Month		Year
Interviewer's name			Signature	
Supervisor's name			Signature	

Part I: Socio-demographic characteristics

S.No	Variable	Response	Skip to
Q01	Age of child?	………..Month	
Q02	Sex of child?	1. Male 2. Female	
Q03	Religious of child?	1. Orthodox<br>2. Muslim<br>3. Protestant<br>4. Catholic<br>5. Other (specify)……………..	
Q04	Sex of care giver?	1. Male<br>2. Female	
Q05	What is the marital status of care giver?	1. Married<br>2. Divorced<br>3. Widowed	

		4. Single<br>5. Other (specify)____________	
Q06	What is the educational level of care giver?	1. Unable to read and write<br>2. Able to read and write<br>3. Grade 1-8<br>4. Grade 9-12<br>5. College and above	
Q07	What is the current occupational status of care giver relation to child?	1. Herder<br>2. House wife<br>3. Merchant<br>4. Farmer<br>5. Private employee<br>6. Government employee<br>7. Other (specify) _________	
Q08	Sex of household head?	1. Male<br>2. Female	
Q09	Number of individuals in the house hold?	1. Two<br>2. Three<br>3. Four<br>4. Five and above	
Q10	Place of residence?	1. Urban<br>2. Rural	
Q11	Monthly income of care giver?	1. < 750 ETB<br>2. 750-1500 ETB<br>3. > 1500 ETB	

Part II Feeding related factor

Q12	Do you practice dietary diversity during food preparation?	1. Yes<br>2. No	

Q13	Which product use in dietary diversity?	1. Animal product<br>2. Plant product<br>3. Both	
Q14	When start introduction of complementary foods?	1. < or = 6 months<br>2. > 6 months	
Q15	Duration of breast feeding practice?	1. < 6month<br>2. 6-12month<br>3. 1-2year<br>4. Above 2years	
Q16	Do you have nutritional knowledge?	1.Yes<br>2. No	
Q17	In the past four weeks, did you worry that your household would not have enough food? (Food insecurity)	1.Yes<br>2. No	
Q18	A child does take animal products (meat, egg milk…?)	1. Yes<br>2. No	
Q19	Meal frequency per day?	1. One times<br>2. Two times<br>3. Three times<br>4. Four times and above	

Part III: Health Care and Diseases Characteristics

**No_**	**Question**	**Response**	**Skip to**
Q20	Recent history of acute blood loss in past 4 week?	1. Yes<br>2. No	
Q21	History of recent blood transfusion	1. Yes	

	reaction?	2. No	
Q22	History of recent surgical procedure?	1. Yes<br>2. No	
Q23	Recent history of accident?	1.Yes<br>2.No	
Q24	History of intestinal protozoan infections (Amoebasis, Giardiasis) in past weeks?	1. Yes<br>2. No	
Q25	History of soil-transmitted helminthic infection?	1. Yes<br>2. No	
Q26	History of malaria infection?	1. Yes<br>2. No	
Q27	Recent history of epistaxis?	1. Yes<br>2. No	
Q28	History of Chronic diseases?	1. Yes<br>2. No	

Annex III: Laboratory report form

Serial no__________

Date of specimen collection: __________ time: _____________

Blood examination

- CBC examination report
